# Mini-laparotomy for cholecystectomy in resourced limited settings: a 10-year retrospective hospital-based study

**DOI:** 10.11604/pamj.2022.42.304.32681

**Published:** 2022-08-23

**Authors:** Samir Ismail Bashir, Rayan Bakari Adam Mohamed, Khalid Abbas Owish, Abdalla Mohamed Abdalla, Abdullah Misbah Abdullah, Yasir Babiker Ali

**Affiliations:** 1 University of Bakht Al-Ruda, Faculty of Medicine and Health Sciences, Department of General Surgery, Bakht, Sudan,; 2 Department of Physiology, University of Bakht Al Ruda, Faculty of Medicine and Health Sciences, Bakht, Sudan,; 3 Trauma & Orthopedic Department, Omdurman Islamic University, Khartoum, Sudan

**Keywords:** Cholecystectomy, developing country, mini-laparotomy

## Abstract

Cholecystectomy is a commonly performed abdominal procedure, the gold standard currently is the laparoscopic approach and, however, the facilities and expertise for laparoscopy are not available widely, especially in developing countries. A Mini-laparotomy cholecystectomy is an additional approach that is performed through an incision that is less than 5 cm thus minimizing the complications of the traditional open cholecystectomy and the postoperative hospital stay. The study aims to evaluate the outcome of mini-laparotomy cholecystectomy in terms of operative duration, complications, and hospital stay in a rural hospital. This is a retrospective study conducted in El-Dwaim Teaching Hospital, Sudan. All cases of mini-laparotomy cholecystectomy conducted from March 2009 to December 2020 were included and retrospectively studied. Descriptive statistics were applied using SPSS version 25. A total of 512 mini-laparotomy cases were involved in the study, of those 442 aged more than 40 years. The operation lasted less than 60 minutes for 486 of the participants, and the most frequent cholecystitis complication observed intraoperatively was mucocele, occurring in 70 (13.6%) participants. Intraoperative complications due to mini-laparotomy occurred in 4 (0.8%) cases, in the form of bleeding and none of the observed cases converted into open cholecystectomy. Postoperative complications occurred in the form of wound infection in 7 participants, biliary leak in 1 participant, and fistula formation in 1 participant. Post-operative hospital stay was 24 hours for 458 participants. Mini-laparotomy cholecystectomy is a safe minimally invasive approach with low rates of complications and short postoperative hospital stay, making it an optimum approach in facility-deprived countries.

## Introduction

Cholecystectomy is one of the most commonly performed abdominal procedures, in the United States, 1.5 million cholecystectomies are performed annually [1]. Before the year 1991, the procedure was standardly performed through an open abdominal incision (Laparotomy), which usually required 2 to 6 days of postoperative hospital stay, however afterward, given the advances in laparoscopic surgery the gold-standard changed to laparoscopic cholecystectomy [2], currently, 92% of all cholecystectomies are performed through this approach.

In developing countries like Sudan, open cholecystectomy is still widely practiced among rural hospitals due to the high cost of laparoscopic cholecystectomy, technical limitations, and lack of qualified professionals for this procedure. In the conventional open procedure, the gallbladder is approached through an 8-10 cm incision with considerable complications, tissue damage, pain, and hospital stay. Mini laparotomy cholecystectomy is considered a minimally invasive technique and is widely practiced in rural hospitals with limited resources. This operation is done through an incision that is less than 5 centimeters in length, and thus it minimizes the complications associated with open cholecystectomy (wound infection, hematoma, or incisional hernia) and was found to have a shorter duration of hospital stay compared to the traditional open cholecystectomy [3,4].

Both mini-laparotomy and laparoscopic cholecystectomies have no significant difference in terms of hospital stay and postoperative morbidity, with laparoscopic surgeries having a longer Intraoperative duration [5]. A recent meta-analysis of randomized controlled trials found that laparoscopic cholecystectomies have a longer operative duration when compared to mini-laparotomy cholecystectomy while resulting in similar outcomes in terms of hospital stay, intra-operative and post-operative complications [6]. This study aims to evaluate mini-laparotomy cholecystectomy as the most suitable and feasible technique in developing countries with limited resources likewise Sudan.

## Methods

**Study design and study area:** this is an observational, retrospective study conducted in El-Dwaim Teaching Hospital, Sudan. All cases of mini-laparotomy cholecystectomy conducted from March 2009 to December 2020 were included and retrospectively studied. El-Dwaim Teaching Hospital is located in El-Dwaim, a rural region located in the White Nile State, Sudan. This hospital provides training for medical students, house officers, and registrars, in addition to providing services for an estimated 450,000 individuals.

**Operation:** the operations were conducted under aseptic conditions, and under general anesthesia with muscle relaxants and intubation. Intra-operative prophylactic antibodies were administered at the induction of anesthesia for all of the cases, in the form of ceftriaxone and metronidazole. A 4-5 centimeters mini-Kocher incision, 1-2 fingers below the costal margin was done. Using sharp dissection, the skin, subcutaneous fat, and the anterior rectus sheath were opened and the muscles were divided. Retractors were then inserted to visualize the operation scene, and a lap pad was placed in the sub-hepatic space for operative exposure and isolation of the operative field. Dissection of the structures in Calot's triangle was then carried out, the cystic duct was transected using double ligature followed by ligation of the cystic artery and in some cases, mass ligation was done. The stump was ligated using a non-absorbable suture. In case of complications (bleeding, empyema) a vacuum-suction was placed in the gallbladder fossa. Post-operative pain relief was granted by non-steroidal anti-inflammatory drugs (diclofenac 75 mg, intramuscularly) twice daily on the first post-operative day and as requested by the patients thereafter. All the participants received post-operative antibiotics for duration of 5 to 7 days.

**Data analysis:** it was reviewed, ordered, and coded, and then the Statistical Package for the Social Sciences (SPSS) version 25 was used for data analysis. Simple descriptive statistics were applied to analyze the participant's characteristics, preoperative morbidities, duration of the operation, Intraoperative and postoperative complications, and postoperative hospital stay.

## Results

### Age, clinical presentation and co-morbidities

A total of 512 mini-laparotomy cases were involved in the study, of those, 442 were aged more than 40 years (Table 1). There were 363 females (70.89%) and 149 males (29.1%). The most frequent clinical presentations are outlined in Table 2. The commonest complaint was biliary colic (n=215, [41%]). Table 3 shows Medical co-morbidities present among the study participants, they were mainly in the form of hypertension (75 participants [14.6%]), diabetes (62 participants [12.1%]), or both (21 participants [4.1%]).

**Table 1:** age groups and gender in the study

Age	N (%)
<30 years	18 (3.5)
30-40 years	52 (10.2)
>40 years	442 (86.3)
**Gender**	
Male	149(29.1%)
Female	363(70.89%)

**Table 2:** clinical presentation of patients in this study

Presentation	N(%)
**Biliary colic**	215(41%)
**Recurrent cholecystitis**	127(24.8%)
**Dyspepsia**	58(11.3%)
**No biliary symptoms**	112(21.8%)

**Table 3:** preoperative medical co-morbidities

Co-morbidity	N(%)
**Hypertension**	75 (14.6)
**Diabetes**	62 (12.1)
**Hypertension and diabetes**	21 (4.1)
**No Co morbidities**	354 (69.1)

### Intraoperative findings and complications

Table 4 shows the intraoperative findings and complications. The duration of the operation ranged from 30 to 90 minutes, and in 486 participants it lasted less than 60 minutes. There were no complicated cholecystitis findings in 392 (76.6) participants. The most frequent complication of cholecystitis observed was mucocele, occurring in 70 (13.6%) participants, followed by adhesions in 42 (8.2%). Intraoperative complications due to mini-laparotomy cholecystectomy occurred in 4 (0.8%) participants and were in the form of bleeding. None of the conducted operations were converted to open cholecystectomy.

**Table 4:** Intra-operative findings and complications

Category	N (%)
**Adhesions**	42 (8.2)
**Mucocele**	70 (13.6)
**Empyema**	4 (0.8)
**Bleeding**	4 (0.8)
**No complications observed**	392 (76.6)
**Conversion to open cholecystectomy**	0 (0.0)
**Median Duration of Operation**	60 minutes (range: 30-90 minutes)

### Postoperative complications

For 502 participants (98.0%) there were no postoperative complications (Table 5). The most frequent complication was wound infection, occurring in 7 (1.4%) patients. One patient (0.2%) died postoperatively, secondary to hepatic failure, however, this patient had pre-existing active hepatitis B.

**Table 5:** post-operative complications

Category	N (%)
**Wound infection**	7 (1.4)
**Biliary leak**	1 (0.2)
**Fistula formation**	1 (0.2)
**Hepatic Failure (death)a**	1 (0.2)
**No complications observed**	502 (98.0)

a: The patient had pre-existing hepatitis B

### Post-operative hospital stay

Table 6 shows the duration of postoperative hospital stay. For 458 (98.4%) the postoperative hospital stay was 24 hours.

**Table 6:** duration of post-operative hospital stay

Category	N (%)
**24 hours**	458 (89.4)
**72 hours**	52 (10.2)
**2 weeks a**	2 (0.4)

a: In those two patients, the post-operative hospital stay was more than 2 weeks due to post-operative complications (bile leak, wound infection)

## Discussion

The concept of mini-laparotomy cholecystectomy was introduced in the year 1982 by the surgeons Dubois and Berthelot. As the popularity of the minimally invasive surgery concept grew in the early 1990s so did mini-laparotomy cholecystectomy, initial results supported this approach, as it provided the same outcome as the traditional open (large incision) cholecystectomy with fewer: operative duration, complications, and hospital stay [3,7]. This study aimed to evaluate mini-laparotomy cholecystectomy, in terms of operative duration, Intraoperative complications, postoperative complications, and hospital stay. For this purpose, a total of 512 mini-laparotomy cholecystectomies conducted from March 2009 to December 2020 in El-Dwaim Hospital, Sudan, were collected and retrospectively studied. In this study, bleeding occurred in 4 (0.8%) patients, wound infection occurred in 7 (1.4%) patients, biliary leak in 1 (0.2%) patient, and hepatic failure in 1 (0.2%) who subsequently died, however, he had a history of active hepatitis B. A similar study evaluating mini-laparotomy in a rural area in southern Africa found comparable results, out of 248 patients involved in their study, bile leaks occurred in 6 (2.4%) patients, bleeding occurred in 1 (0.4%), wound infection occurred in 7 (2.8%) patients, Incisional hernia occurred in 8 (3.2%) patients [8]. this high frequency of wound infection could be attributed firstly to the skin incision, and the subsequent retraction, which may predispose to tissue injury and facilitate the occurrence of infection [9].

Common Bile Duct injury, which is regarded as the most feared complication of cholecystectomy and is consistency with a previous study didn´t occur in any of the involved participants [8]. Since the introduction of laparoscopic cholecystectomy, the rate of those injuries increased and currently ranges from 0.3% to 0.4%. An additional finding of this study is that none of the involved cases converted to open cholecystectomy, previous studies however reported conversion rates. The conversion rate of mini-laparotomy cholecystectomy ranged from 3.7% to 16% in previous studies [8,10]. The median duration of the operation was 60 minutes (range: 30-90 minutes). The duration is comparable with that of previous studies, where the median operative operation time was 40 minutes [8] and 35 minutes [9] and in a prospective study assessing mini-laparotomy cholecystectomy the mean operative time was 66.8 minutes [10]. Compared to laparoscopic cholecystectomy, multiple previous studies confirmed that the laparoscopic approach got a longer operative duration compared to the mini-laparotomy approach [5-7].

For most of the patients, the post-operative admission period was 24 hours (486 patients), furthermore, 52 participants (10.2%) had a post-operative admission period of 72 hours. Two patients (0.4%) were admitted for two weeks; however, this was due to the presence of postoperative complications (wound infection and bile leak). A similar study found comparable results; the median length of hospital stay was 48 hours [8]. A laparoscopic approach is superior to the mini-laparotomy approach in having a shorter duration of hospital stay and a more rapid return to work [3], however, in this study, the duration of hospital stay is highly approximate.

Despite the presence of complications in this study and a relatively long hospital stay, the mini-laparotomy approach is superior to the Laparoscopic approach in the absolute absence of pneumoperitoneum; easier extraction of the resected gallbladder; isolation of the operative field easily; and the possibility of performing it even in cases of the presence of multiple previous abdominal operations or adhesions, in this study, adhesions were present in 42 (8.2%) of the patients, nevertheless, the operation was carried out and the outcome was nearly perfect for all the patients, this wouldn´t have been the case if the laparoscopic approach was adopted [10], and thus, the mini-laparotomy approach could be the wiser approach not only in the absence of suitable facilities but also when the cholecystitis is complicated or adhesions are expected. Limitations of this study firstly include the single-centred nature of the study, as it was performed in a single hospital, thus the study findings are less generalizable. Secondly, occupational status and return-to-work time were not assessed, which could´ve served as an additional means to evaluate mini-laparotomy cholecystectomy.

## Conclusion

In conclusion, the mini-laparotomy approach for cholecystectomy is a safe, minimally invasive approach, with very low rates of Intraoperative and postoperative complications, and a short duration of post-operative hospital stay. And can be considered a time-saving, cost-effective, and feasible technique in rural hospital

### What is known about this topic

- Cholecystectomy is one of the most commonly practised procedures; the last decades characterized by the growth of minimally invasive techniques in the surgical management of gall bladder disease, which includes laparoscopic and mini-laparotomy cholecystectomy;
- A laparoscopic approach is considered as the gold standard procedure, however open cholecystectomy is still widely performed in developing countries due to the lack of sufficient facilities, expertise, and high expense.

### What this study adds

- Mini laparotomy cholecystectomy is a modified approach for conventional open cholecystectomy; it has a similar outcome to laparoscopic surgery in terms of duration of surgery, hospital stay, and complications;
- It can be considered as a suitable alternative for the laparoscopic approach in rural hospitals where healthcare resources are extremely low, and patients cannot afford the expense of laparoscopic surgery.
